# The Duty of Hospitals in Regard to Thrift

**Published:** 1908-07-04

**Authors:** 


### NURSING ADMINISTRATION.

### THE DUTY OF HOSPITALS IN REGARD TO THRIFT.

There is no more delightful hobby known to man than saving money. When people talk of thrift as though it were an unpleasant virtue, they forget that love of money has been quoted by the philosophers from time immemorial as the root of all evil, and that so far from feeling compelled to exhort men or women to love their gains, the social reformer in days not so very long gone by was chiefly occupied in trying to open their purse strings and avoid the perils of avarice. With the exception of a few wasteful persons, of weak intellect, all wage-earners are tinged with the healthy appetite for saving. Some do it to obtain an immediate and tangible benefit, for the annual holiday, or to make a handsome present. Some do it from a distaste for spending money, some from a sheer love of coin, and others from a due sense of proportion in life's adventures and with a clear vision of future responsibilities. It is of the utmost importance for every profession that its members should early be led to turn this agreeable taste in a direction likely to bring them the best possible advantage. No profession can be strong unless its members are secure from want and dependence during their entire career. Therefore it directly interests all employers that the surplus earnings of the units which make up the whole body should be safely disposed. It is matter of common observation that nurses are exposed to peculiar dangers in respect of their savings. Among the innumerable instances of distress in elderly nurses which have come under the notice of the present writer, no single one has been produced from want of thrift. Invariably there is proof of efforts consistently made to lay by for the future, but disaster has been caused by the wrong disposition of the little hoard. For nurses, being entirely unversed in business matters, have always fallen a ready prey to bad investments, and needed sorely the Insurance Fund adapted to their own particular needs which is now celebrating its twenty-first year of existence in conspicuous success.

Do hospital managers throughout the country recognise their duty in respect of this matter to the nurses who pass through their establishments? It is a little disheartening to glance through the published returns and notice how few hospitals have affiliated to the Royal National Pension Fund for Nurses for the benefit of their staff. By the admirable system of federation offered by the Fund hospitals are enabled to secure all members on the staff from want in illness and old age. It must be remembered that after the first few years every additional year spent in hospital renders it a little more difficult for the nurse to get remunerative work elsewhere. Hence the importance of supplying a counter-balance by setting a premium on long terms of service such as is offered by affiliation with the Fund. But under this system the nurse is not only safeguarded during the time she is in hospital, and assisted to provide for her future, she is also taught how to make the best possible disposition of her own savings, and this habit once established is not likely to be abandoned. Ultimately the burden of making proper provision for her future must rest upon the individual nurse. But the burden of helping her to do this in the right way, of teaching her how to save and where her savings will be safe, is one which the hospitals ought to be prepared to assume. Nurses come to them as young women without any previous business experience, often without friends able to advise them wisely. It rests with the authorities, and in particular with the matron, to instil into the staff the necessity for starting their finances on a good principle, and this can never be so successfully done as in the institutions where nurses are encouraged by a process of gentle compulsion to make their deposit in the Royal National Pension Fund for Nurses. The two main requisites for an insurance fund for nurses—security and elasticity—have been nobly met in the Fund. That the men to whom the management was entrusted would see to it that the nurses' money was safe was from the first beyond question. But it was not so clear at the commencement that the managers would realise as they have done the necessity for an elastic treatment of the objects of the Society. There are no hard and fast rules about paying money on particular dates, for it is well understood that nurses often receive their money irregularly, and have no safe place in which to keep it. The Secretary will receive just such sums as the nurse can afford to pay, and whenever she likes to pay it, and will apply it for the payment of premiums as the nurse shall direct. Every pound paid in becomes a nucleus of capital which may in emergency prove of the highest service to its possessor. Such emergencies are liable to enter into the lives of all workers, and the way out of them often needs the golden key. But not for that reason is the nurse compelled to surrender the policy which is to bring her independence in the future. She can procure a loan on its value, and repay it by degrees at a low rate of interest, with the hope before her of regaining her former good position. If a period of ill-health or bad fortune intervenes, and the premiums are not forthcoming in any given year, every consideration is shown, for the authorities are well versed in the fluctuations of a nurse's earnings, and give facilities for making up the payments when affairs begin to brighten. Thus the Pension Fund nurse is freed from the dangers of the money-lender, and from the terror of feeling that she is left alone to struggle unaided in a world of keen business people, all eager to over-reach her. She has her own business adviser, and one who in crushing misfortune can command powerful aid on her behalf. It is the highest benefit which any hospital can bestow on the members of the staff to introduce the nurses to this great source of strength and protection. We earnestly commend the matter to the attention of matrons, in the hope that, even where no system of federation can be carried out, the nurses may early learn by experience to understand that there is no safer or more enjoyable way of saving than under the auspices of the Royal National Pension Fund for Nurses.

